# Before the task begins: linking resting-state gamma oscillations to face-name associative memory

**DOI:** 10.1007/s11571-026-10551-y

**Published:** 2026-09-15

**Authors:** Gaetano Scianatico, Giorgia Francesca Scaramuzzi, Ece Çağlayan, Anna Concetta Spina, Ester Cornacchia, Volker Thoma, Paolo Taurisano, Davide Rivolta, Valerio Manippa

**Affiliations:** 1 Department of Education, Psychology and Communication, University of Bari Aldo Moro, Bari, Italy; 2 Department of Translational Biomedicine and Neuroscience, University of Bari Aldo Moro, Bari, Italy; 3 Department of Health Informatics, Graduate School of Informatics, Middle East Technical University (METU), Ankara, Turkey; 4 Department of Psychological Sciences, School of Psychology, University of East, London, UK; 5 Department of Precision and Regenerative Medicine and Ionian Area, University of Bari Aldo Moro, Bari, Italy; 6 Department of Theoretical and Applied Sciences, eCampus University, Novedrate, CO Italy

**Keywords:** Spontaneous brain activity, Cross-modal memory, Cognition, Resting-state connectivity

## Abstract

Assessing associative memory, particularly in complex paradigms such as face-name association, enables monitoring of cognitive functioning. Electrophysiological correlates of associative memory could provide fundamental insights into the functional role of brain dynamics. This study explored whether resting-state EEG (rsEEG) spectral power and coherence were associated with performance on the Italian Face-Name Association Test (ItFNAT). Thirty-two healthy adults (*M*_age_ = 23.28) underwent an eyes-closed rsEEG recording before the administration of the ItFNAT, which provides scores for immediate recall (IR), delayed free recall (DFR), and delayed total recall (DTR). Frontal, temporal, central and posterior rsEEG spectral power density and theta and gamma network coherence were analyzed in relation to the test scores. High-gamma power (h-γ, 51–100 Hz) was significantly associated with DTR performance, explaining approximately 38% of the adjusted variance. Frontal and temporal h-γ power were positively associated with DTR performance, whereas posterior h-γ power showed a negative association. Furthermore, low-gamma coherence (l-γ, 30–49 Hz) showed trend-wise negative associations with delayed-recall performance. Spontaneous gamma activity showed region-specific associations with face-name memory performance in healthy adults, involving frontal, temporal, and posterior regions. This regional pattern may represent a neurophysiological correlate of individual differences in associative memory retrieval.

## Introduction

Brain oscillations are rhythmic variations in the amplitude and frequency of cortical activity, non-invasively observed and recorded using electroencephalography (EEG), that reflect the fundamental cognitive and sensory processes of both healthy and pathological brain functioning (Ishii et al. 2017; Fernández-Rubio et al. 2025; Grent-‘t-Jong et al. 2016; Palmisano et al. 2025; Rivolta et al. 2015). Among higher-level cognitive domains, memory has been strongly linked to specific patterns of neural dynamics. In particular, theta and gamma activity have been identified as most prevalent in brain areas critical for memory and learning, including the hippocampus and prefrontal cortex (Cornwell et al. 2014; Takeda et al. 2025; Vinnakota et al. 2025). These findings have motivated the use of resting-state EEG (rsEEG) to characterize spontaneous neural dynamics and examine their association with individual differences in memory performance. For instance, Manippa et al. (2025b) observed that immediate and delayed recall performances were positively associated with high-gamma activity (51–100 Hz) in the frontal and temporal regions and negatively correlated with activity in central and posterior regions; furthermore, temporal low-gamma activity (30–49 Hz) was positively associated with delayed recall. These regional findings provided the main empirical basis for the gamma-power expectations examined in the present study. The present work represents the next step of this research program, moving from domain-general memory to the more specific domain of associative memory. Whereas Manippa et al. (2025b) investigated composite memory performance across multiple neuropsychological tests, the current study examines the neural correlates of face-name associative memory using the Italian Face-Name Association Test (ItFNAT).

Within the broader domain of memory, the study of associative memory provides valuable insights into cognitive functioning and enables the early detection of cognitive decline (Macoir et al. 2024). The ability to associate a name with a face is a complex process that requires the integration of episodic memory, visual processing, and verbal retrieval. In the domain of learning and memory, multisensory training and cross-modal binding have been shown to facilitate accelerated learning and enhance performance, even in unimodal tasks (Murray and Shams 2023). However, this ability is often compromised during physiological aging and, more markedly, in the early stages of neurodegenerative disease, possibly due to reduced hippocampal activation (O’Brien et al. 2010). This decline has prompted numerous researchers to design and standardize face-name associative memory paradigms (Alegret et al. 2020; Flores-Vázquez et al. 2023; Manippa et al. 2025c; Rentz et al. 2011, 2023; Troyer et al. 2011), which have demonstrated their efficacy in detecting the progression of cognitive decline, including Alzheimer’s Disease.

Oscillatory activity does not operate in isolation. Associative memory formation and retrieval depend on interactions among distributed neural populations, which can be characterized through measures of functional connectivity such as spectral coherence (Uhlhaas and Singer 2012; Grent-‘t-Jong et al. 2018b). At the anatomical and functional level, face-name associative memory involves frontal, temporal, and parietal systems contributing to crossmodal integration, semantic binding, cognitive control, and episodic retrieval (Murray and Shams 2023; Ramanan et al. 2019). However, these studies do not identify a specific EEG frequency band underlying each of these networks. Frequency-specific support for gamma connectivity comes from electrophysiological studies of associative learning and memory. Miltner et al. (1999) reported increased gamma-band coherence between cortical regions processing the stimulus categories involved in an associative-learning procedure. Frontoparietal high-frequency coherence has also been observed during short-term memory processing, including bidirectional frontoparietal information flow in the gamma range (Babiloni et al. 2004). Furthermore, gamma-range frontoparietal connectivity has been reported during episodic emory encoding and retrieval, with task- and hemisphere-dependent variations (Babiloni et al. 2006). These findings support gamma as a frequency range of interest for memory-related interregional communication.

The present study investigates how variations in oscillatory activity and coherence in the theta, low-gamma, and high-gamma bands relate to performance on the Italian Face-Name Association Test (ItFNAT; Manippa et al. 2025c), a cross-modal associative memory test. The division of gamma into two sub-bands is consistent with recent research suggesting distinct contributions of slower and faster gamma oscillations to memory-related processes (e.g., Colgin 2015; Kucewicz et al. 2017). Based on previous observations (Manippa et al. 2025b), we expected delayed recall performance to be positively associated with spontaneous high-gamma activity in frontal and temporal regions and with temporal low-gamma activity, whereas the analysis of theta activity was purely exploratory. Furthermore, for exploratory purposes, we investigated the relationship between resting-state theta and gamma coherence and ItFNAT performance.

## Materials and methods

### Participants and procedure

**Fig. 1:**
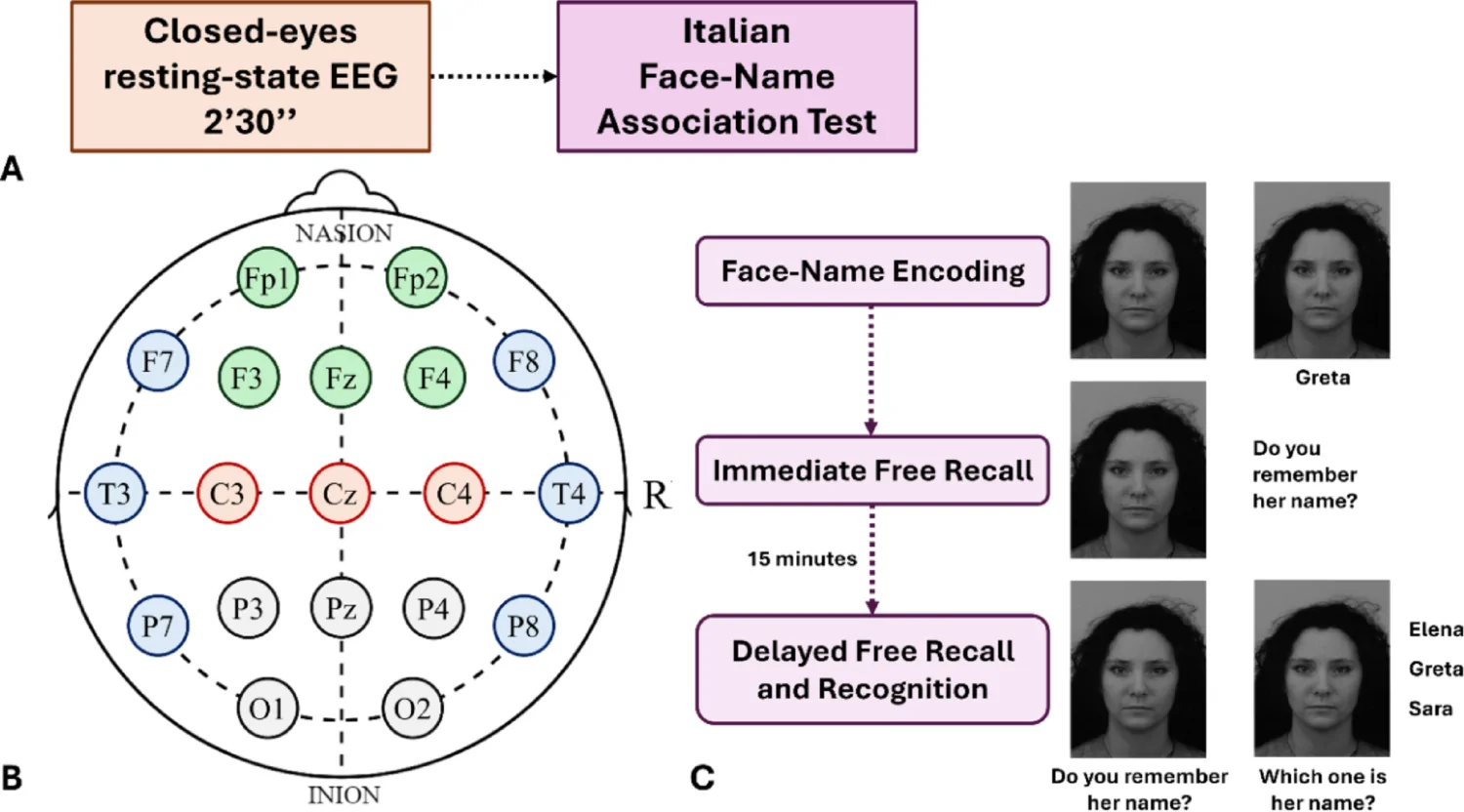
**A** Experimental Protocol. **B** Electroencephalography (EEG) montage and Regions of Interest (ROI). Green electrodes represent the Frontal ROI, red electrodes represent the Central ROI, blue electrodes represent the Temporal ROI, and gray electrodes represent the Posterior ROI. **C** Italian Face-Name Association Test Protocol: consisting of encoding phase (learning faces and names), immediate recall (naming faces after brief exposure), and delayed recall (retrieval after 15 min, with recognition trials if needed)

Thirty-two (19 females, 13 males; 30 right-handed, 2 left-handed) young adults (*M*_age_ = 23.28, *SD*_age_ = 2.69, *M*_edu_*=* 16.04, *SD*_edu_*=* 2.48) without any history of neurological or psychiatric conditions were recruited from local universities and communities using a snowball sampling procedure. Participants first provided informed consent and completed the Italian version of the Edinburgh Handedness Inventory (Oldfield 1971; Salmaso and Longoni 1985) and the online short version of the Cognitive Reserve Index (CRI, *M* = 97.98, *SD* = 7.18) questionnaire (Mondini et al. 2023). The study adhered to the ethical standards of the World Medical Association Declaration of Helsinki, and the protocol received approval from the Ethics Committee of the University of Bari “Aldo Moro” (protocol number: ET-24–01). Twenty-four participants had also taken part in a previous study from the same research project (Manippa et al. 2025b). Although the two studies partially shared participants, the present analyses were based on a different EEG dataset (i.e., a single pre-task resting-state recording rather than the average of two recordings acquired one week apart) and addressed different outcomes. The experiment consisted of a 2-minute and 30-second eyes-closed rsEEG recording followed by an associative memory evaluation using the ItFNAT (Manippa et al. 2025c) (see Fig. 1).

### Memory assessment

Associative memory was assessed using the Italian Face-Name Association Task (ItFNAT; Manippa et al. 2025a, c), which required participants to learn and recall associations between faces and names. The ItFNAT includes three sequential phases: (i) in the encoding phase, participants viewed 16 faces for 2 s each and were then asked to read aloud the names and memorize the corresponding face-name pairs; then, (ii) in the immediate recall phase, each face was presented and participants had up to 10 s to recall the correct name; after their response, the proper name was displayed again for reinforcement; finally, (iii) in the delayed recall phase, which began after 15 min filled with unrelated tasks (i.e., a computerized version of the Stroop test), participants were presented with the faces again and asked to freely recall the associated names; if they failed, a recognition task followed with three choices: the correct name, a previously seen but incorrect name (interference), and a new name (intrusion). Scoring was based on correct immediate recall (IR), delayed free recall (DFR), and delayed total recall (DTR; including free recall and recognition).

### rsEEG data recording and pre-processing

EEG was recorded using a battery-driven portable 19-channel Starstim 20/32 system (Neuroelectrics, Spain) at a sampling rate of 500 Hz. The neoprene head cap was fitted with 19 electrodes according to the international 10–20 system and electrically referenced to a clip electrode placed in the right ear lobe (see Fig. 1B). Electrode impedances were kept below 10 kΩ throughout the recording. EEG was acquired for 150 s by the Starstim software suite (NIC) and pre-processed offline with the EEGlab toolbox in Matlab 2023 (MathWorks Inc., USA).

EEG data were first filtered using a high-pass filter of 1 Hz and a low-pass filter of 150 Hz. A notch filter was set at 50 Hz. Recordings were referenced to the common average of all electrodes, and the Artifact Subspace Reconstruction algorithm (Chang et al. 2018) was used in conjunction with the EEGlab plugin to identify and correct or remove segments exceeding predefined amplitude thresholds. Recordings were then visually inspected to exclude further artifacts that survived. After artifact rejection, the duration of the retained artifact-free EEG data was quantified for each participant. Then, independent component analysis (ICA) was applied to the continuous data to decompose the measured EEG signals into independent components. Those associated with artifacts (i.e., Muscle, Eye, Heart, and Channel Noise) were identified and removed (range 0–4, Mdn = 3 per trace).

The spectral segmentation of the EEG data was then conducted using the Brainstorm toolbox for Matlab (Tadel et al. 2011). Welch’s method was applied by averaging 1 s signal windows, with 50% overlap calculated using the Fast Fourier Transform (FFT) algorithm. Then, the power spectral density (PSD) or periodogram was computed in signal units²/Hz for each electrode across five frequency bands: Theta (θ, 4–7 Hz), Alpha (α, 8–12 Hz), Beta (β, 13–29 Hz), low-Gamma (l-γ, 30–49 Hz), and high-Gamma (h-γ, 51–100 Hz). Finally, EEG electrodes were clustered into four Region of Interests (ROIs; see Fig. 1B) by averaging the activity of the respective electrodes: (i) Frontal (Fp1, Fp2, F3, F4, Fz); (ii) Central (C3, C4, Cz); (iii) Temporal (F7, F8, T3, T4, P7, P8); and (iv) Posterior (P3, P4, Pz, O1, O2). Coherence analysis was performed in Brainstorm using magnitude-squared coherence between electrode pairs within the θ, l-γ, and h-γ bands, using 1-s windows with 50% overlap. Coherence values were averaged across epochs to generate pairwise connectivity matrices. Frequency-dependent electrode-pair clusters were defined a priori, based on anatomically plausible ipsilateral and midline interregional connections within the 19-channel montage (see Table 1). Particularly, for both low- and high-gamma frontal–temporal, temporal–parietal, and frontal–parietal coherence were examined due to their involvement in associative memory encoding and retrieval, while frontal-midline and central–parietal coherence were selected for theta due to its involvement in executive monitoring and mnemonic maintenance (Cavanagh and Frank 2014; Summerfield and Mangels 2005; Tan et al. 2024).

**Table 1:** EEG network clusters, electrode pairs, and frequency bands used for coherence analysis

Network cluster	Electrode couples	Band
Frontal-midline	Fz–F3, Fz–F4, Fz–Fp1, Fz–Fp2, Fz–F7, Fz–F8	Theta
Frontal-temporal	F3–T3, F4–T4, T4-F8, T3-F7	Low-gamma, high-gamma
Frontal-parietal	F3–P3, F4–P4, Pz–F3, Pz–F4, Pz-Fz	Theta, low-gamma, high-gamma
Temporal-parietal	T3–P3, T4–P4, T3–Pz, T4–Pz	Low-gamma, high-gamma
Central-parietal	C3–P3, C4–P4, Cz–Pz, C3–Pz, C4–Pz	Theta

### Data analysis

A G*Power sensitivity analysis was conducted for a fixed multiple-regression model with four predictors, a total sample size of 32, α = 0.05, and statistical power of 0.80. The analysis indicated that the available sample could detect an overall effect of at least f² = 0.444.

Statistical analyses were performed using JASP (version 0.95.4).

Spearman’s rank correlations were calculated among IR, DFR, and DTR to quantify the associations among the three ItFNAT outcomes. Score distributions and the frequency of maximum scores were also inspected to assess potential ceiling effects. Although alpha and beta power were estimated as part of the complete resting-state spectrum, according to our hypothesis, inferential analyses were restricted to theta and gamma bands.

For each ItFNAT score (i.e., IR, DFR, and DTR), three separate multiple linear regression models were constructed, each corresponding to a distinct EEG frequency band: θ, l-γ, and h-γ. Within each model, band power values from four ROIs – Frontal, Central, Temporal, and Posterior – were simultaneously entered as predictors using the enter method, without automated variable selection. Benjamini–Hochberg false discovery rate (FDR) correction was applied to the model-level p-values. In the model remaining significant after FDR correction, multicollinearity among predictors was assessed using the Variance Inflation Factor (VIF) and tolerance values. When moderate collinearity was detected, the respective ROI was excluded, and the regression model was then re-estimated using the remaining variables to improve interpretability and statistical robustness. A nonparametric bootstrap procedure with 5,000 resamples was applied to the models reaching nominal significance before FDR correction to evaluate the stability of the regression estimates. Bias-corrected and accelerated 95% confidence intervals were calculated for the unstandardized regression coefficients. As a post hoc check of the potential influence of unequal artifact-free EEG duration, the duration in seconds of the retained artifact-free EEG data was quantified for each participant. Pearson correlations were calculated between artifact-free EEG duration and the three ItFNAT outcomes. Models that reached significance after FDR correction were also repeated after excluding participants with less than 60 s of artifact-free EEG data. To assess the potential influence of residual electromyographic activity at temporal electrodes, the same model was repeated after excluding the Temporal ROI.

For the coherence analysis, three separate sets of nine Spearman’s rho correlations were computed to examine the relationship between ItFNAT performance and network-specific connectivity. Each set included the three ItFNAT scores (i.e., IR, DFR, and DTR) and coherence values from distinct frequency bands: (i) Frontal-Parietal, Frontal-Midline, and Central-Parietal θ coherence values, (ii) Frontal-Temporal, Temporal-Parietal, and Frontal-Parietal l-γ coherence values, and (iii) Frontal-Temporal, Temporal-Parietal, and Frontal-Parietal h-γ coherence values. To assess the stability of the correlations, 1,000 bootstrap resamples were used to compute 95% confidence intervals for Spearman’s rho. Fisher’s z-transformed effect sizes were also obtained. Benjamini–Hochberg FDR correction was applied separately within each of the three frequency bands, each comprising nine ItFNAT score–coherence correlations. Bootstrap confidence intervals were retained to characterize the uncertainty of the estimates.

## Results

Descriptive statistics for the ItFNAT outcomes were as follows: IR, M = 6.88, SD = 3.78, Mdn = 6.00, IQR = 4.00, range = 1–16; DFR, M = 8.84, SD = 4.02, Mdn = 9.00, IQR = 4.50, range = 2–16; and DTR, M = 14.34, SD = 1.70, Mdn = 14.50, IQR = 2.25, range = 9–16. One participant (3.1%) obtained the maximum IR score, four participants (12.5%) obtained the maximum DFR score, and eleven participants (34.4%) obtained the maximum DTR score. The three ItFNAT outcomes were positively correlated. Spearman’s correlations were ρ = 0.789 between IR and DFR, ρ = 0.775 between IR and DTR, and ρ = 0.675 between DFR and DTR (all *p* <.001). After artifact rejection, an average of 96.19 s (*SD* = 34.00; median = 100.13; range = 29.83–148.25 s) of clean EEG data per session was retained. Artifact-free EEG duration was not significantly associated with IR, *r* =.040, *p* =.827, DFR, *r* =.261, *p* =.148, or DTR, *r* =.187, *p* =.305. Four participants had less than 60 s of artifact-free EEG data. After excluding these participants (*N* = 28), the high-gamma DTR regression model remained significant, F(4, 23) = 7.663, *p* <.001, adjusted R² = 0.497.

The multiple linear regression models to assess whether θ, l-γ, and h-γ rsEEG power (units^2^/Hz) within the four different ROIs predict ItFNAT performance (i.e., IR, DFR, and DTR) are summarized in Table 2.

**Table 2:** Full regression models relating resting-state power spectral density to ItFNAT performance

Frequency bands	ItFNAT Score	Adj. *R*²	F	Model *p*	FDR	ROI	B (coeff.)	β	*p*	BCa 95% CI for B
Theta	IR	0.069	1.576	0.209	0.237					
(4–7 Hz)	DFR	0.068	1.569	0.211	0.237					
	DTR	0.176	2.651	0.055	0.124					
Low gamma	IR	0.103	1.893	0.141	0.212					
(30–49 Hz)	DFR	0.048	1.394	0.263	0.263					
	DTR	0.193	2.849	0.043	0.124					
High gamma	IR	0.245	3.521	0.019	0.086					
(51–100 Hz)										
	DFR	0.112	1.982	0.126	0.212					
	DTR	0.378	5.718	0.002	0.018	Frontal	121.207	0.990	0.006	[45.337, 226.510]
						Temporal	79.419	0.643	0.037	[−17.176, 121.060]
						Posterior	−148.642	−1.163	< 0.001	[−248.917, −61.520]

Adjusted R², F statistics, unadjusted p values, and FDR-adjusted p values are reported for all models

Significant *ROI* region of Interest coefficients and BCa 95% confidence intervals are shown only for models reaching FDR-corrected significance

*IR* immediate recall, *DFR* delayed free recall, *DTR* delayed total recall

Before correcting for multiple comparisons, the regression model using low-gamma power as predictor of DTR was significant (F_4, 27_ = 2.849, *p* =.043, FDR-adjusted *p* =.124, adjusted *R*² = 0.193); similarly, the regression model using high-gamma power as predictors of IR reached significance (F_4, 27_ = 3.521, *p* =.019, FDR-adjusted *p* =.086, adjusted *R*² = 0.245). Finally, the regression model using DTR as the dependent variable and high-gamma power within the four ROIs as predictors remained significant after Benjamini–Hochberg FDR correction F_4, 27_ = 5.718, *p* =.002, FDR-adjusted *p* =.018, adjusted R² = 0.378. The Posterior ROI showed a negative coefficient (β = −1.163, *p* <.001), whereas the Frontal (β = 0.990, *p* =.006) and Temporal (β = 0.643, *p* =.037) ROIs showed positive coefficients. A post hoc follow-up model excluding the Central ROI showed a slightly higher adjusted R², F_3, 28_ = 7.443, *p* <.001, adjusted R² = 0.384, with significant Frontal (β = 0.889, *p* =.008), Temporal (β = 0.475, *p* =.038), and Posterior coefficients (β = −1.156, *p* <.001). In this follow-up model, the 5,000-resample bootstrap yielded BCa confidence intervals excluding zero for all three regional coefficients.

**Fig. 2:**
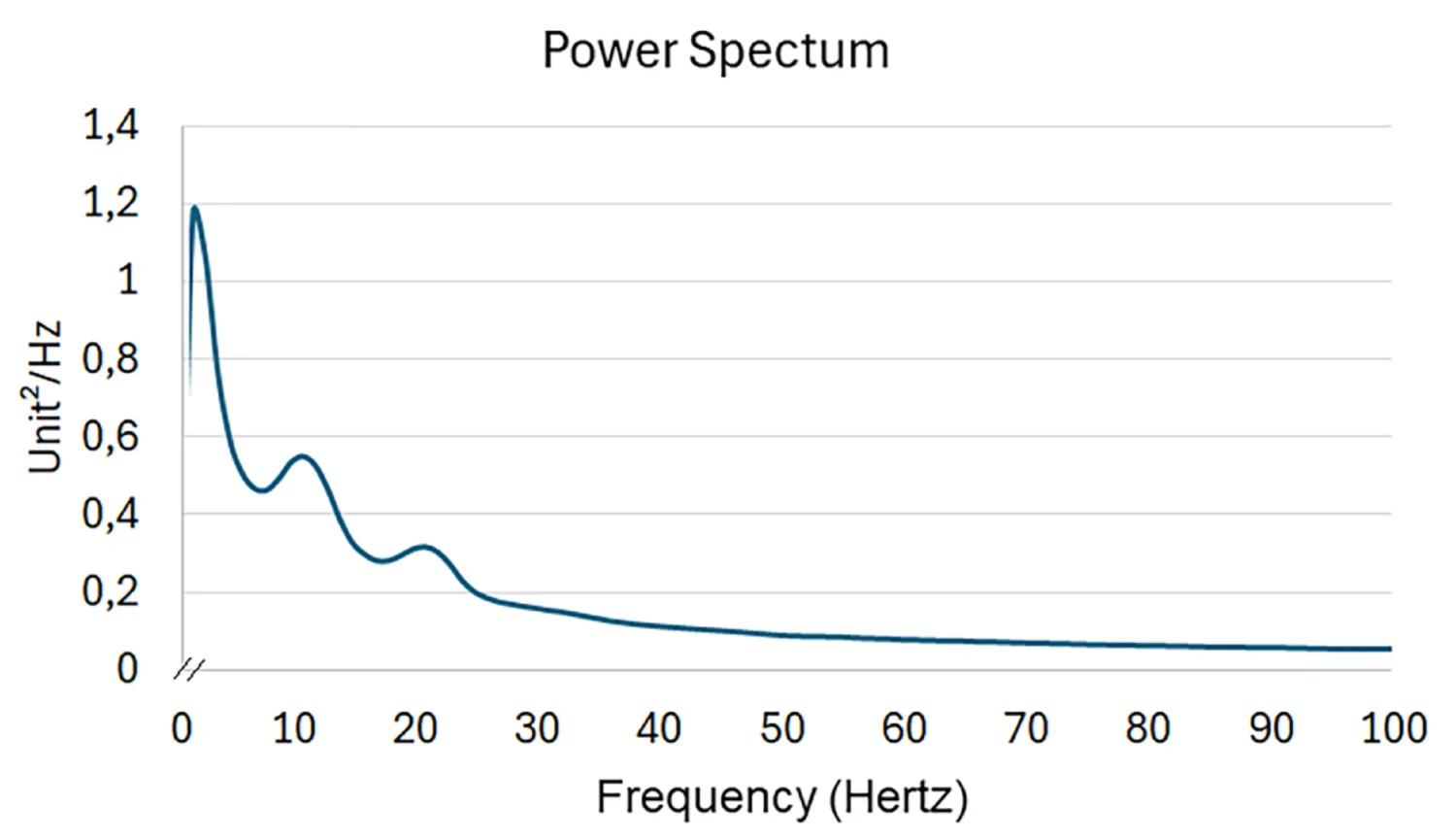
Average resting-state EEG (rsEEG) power spectrum across all participants. The mean spectrum shows a prominent peak around 10 Hz within the alpha band, reflecting typical resting-state alpha activity, and a secondary peak around 20 Hz within the beta band, which may relate to sensorimotor and cognitive idling processes during rest. Beyond these peaks, the spectrum displays the expected slow-to-fast like decline in power across higher frequencies, including the beta and gamma ranges

**Fig. 3:**
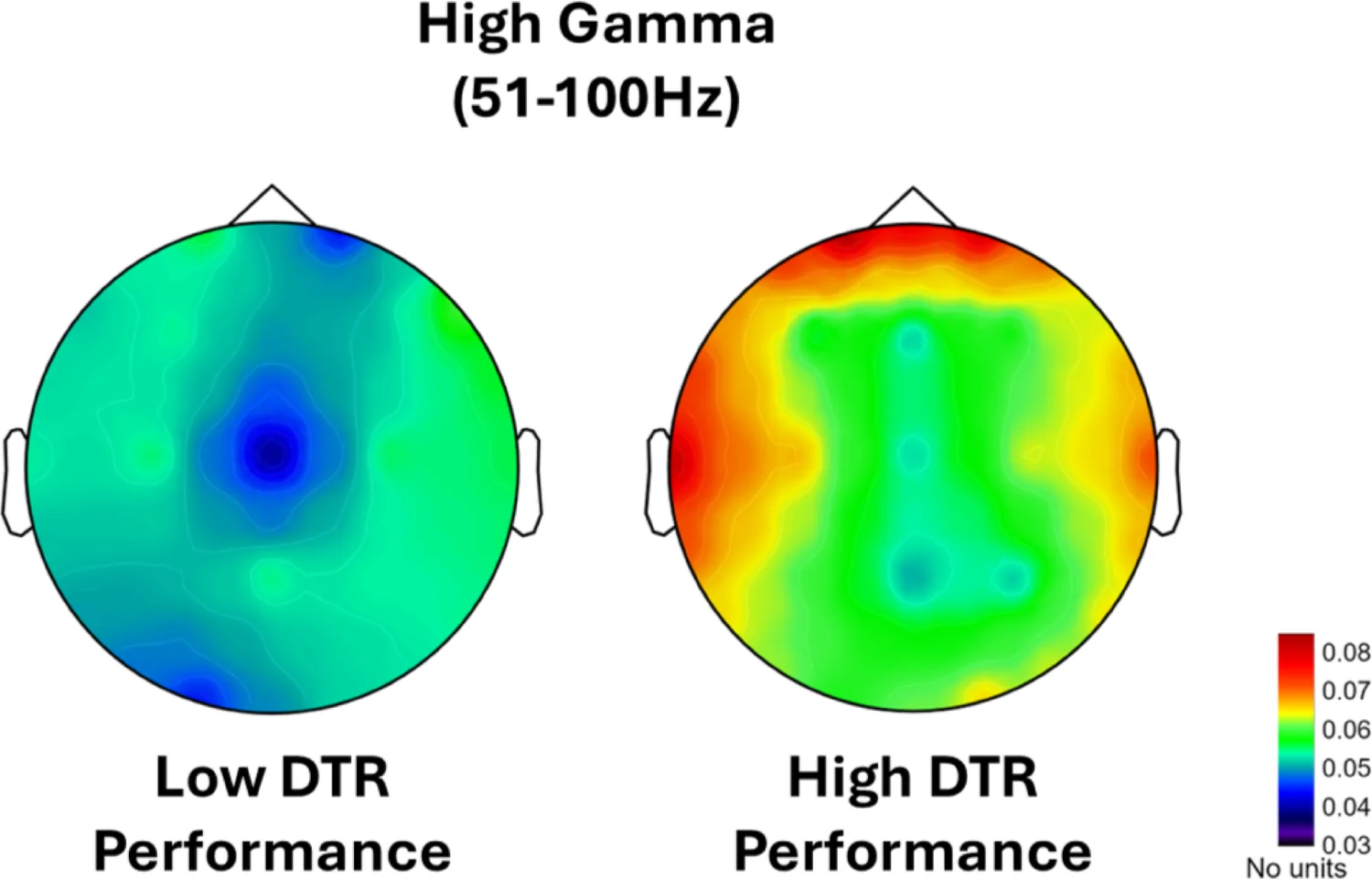
Qualitative topographic representation of resting-state high-gamma power (51–100 Hz; signal units²/Hz) according to Delayed Total Recall (DTR) performance. Each map shows the average rsEEG activity of the four lowest- and four highest-performing participants (2 males and 2 females per group). These subsets were used for visualization purposes only; the figure illustrates the regression pattern reported in Table 3 and is not intended as an independent statistical comparison

Collinearity diagnostics for the significant four-ROI high-gamma DTR model indicated moderate overlap among predictors. Tolerance was 0.178 for the Frontal ROI (VIF = 5.605), 0.195 for the Central ROI (VIF = 5.119), 0.233 for the Temporal ROI (VIF = 4.290), and 0.245 (VIF = 4.086) for the Posterior ROI. The full regression results are reported in Table 2, whereas the post hoc follow-up model is summarized in Table 3. Furthermore, to assess the potential influence of residual electromyographic activity at temporal electrodes, the high-gamma DTR model was repeated after excluding the Temporal ROI. The model remained significant, F_3, 28_ = 5.300, *p* =.005, adjusted R² = 0.294, with a positive Frontal coefficient (β = 1.040, *p* =.007) and a negative Posterior coefficient (β = −1.090, *p* =.001). Thus, the association between high-gamma power and DTR was not exclusively attributable to the Temporal ROI (Figs. 2 and 3).

**Table 3:** Post hoc high-gamma delayed total recall (DTR) regression model after exclusion of the central ROI

Frequency bands	ItFNAT Score	Adj. *R*²	F	Model *p*	ROI	B (coeff.)	β	*p*	BCa 95% CI for B
High gamma	DTR	0.384	7.443	< 0.001	Frontal	108.792	0.889	0.008	[29.776, 200.640]
(51–100 Hz)					Temporal	58.663	0.475	0.038	[5.342, 97.980]
					Posterior	−147.751	−1.156	< 0.001	[−236.205, −59.990]

Adjusted R², F statistics, model-level p value, regional coefficients, and BCa 95% confidence intervals are reported

**Fig. 4:**
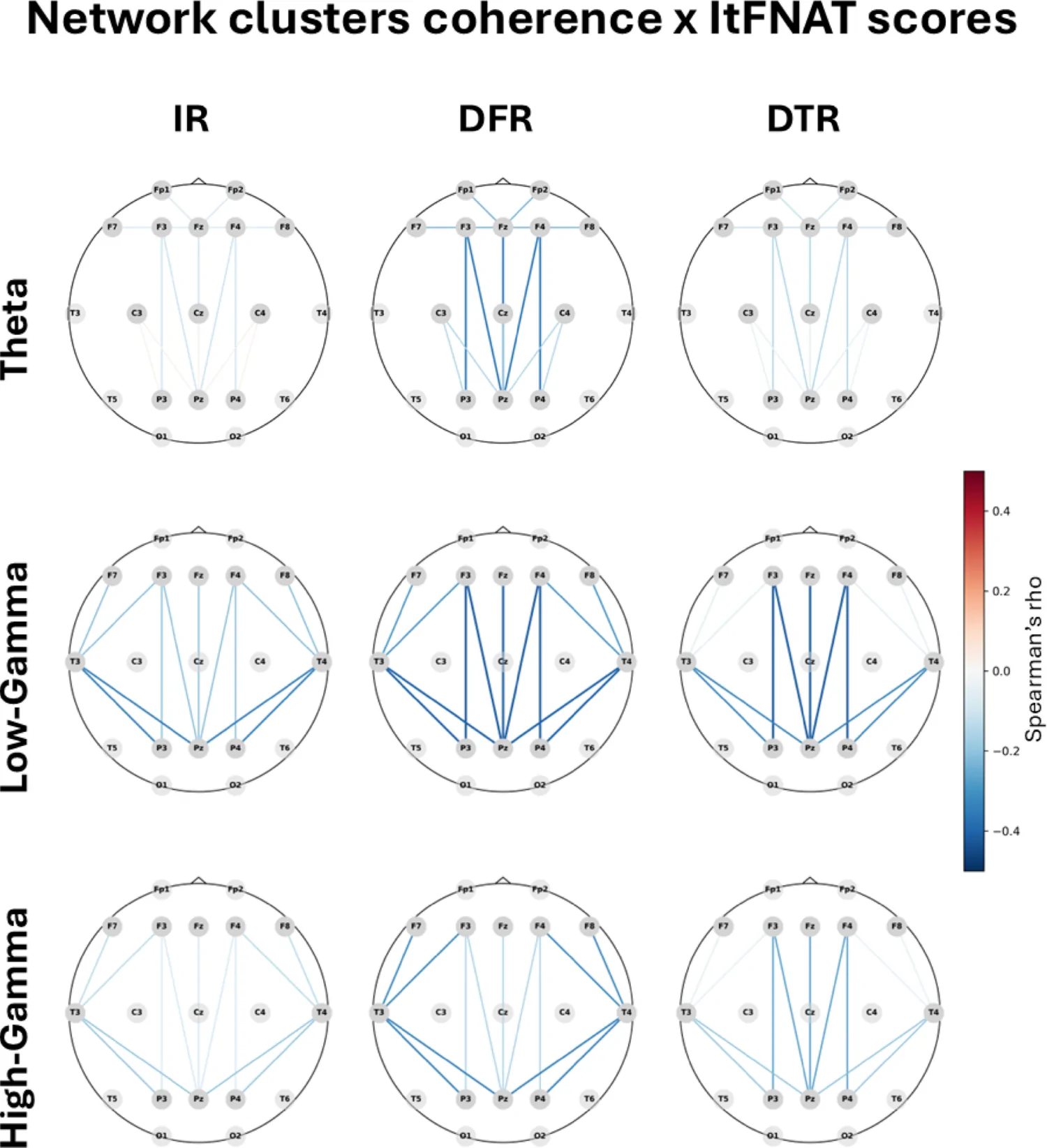
Connectivity maps illustrating Spearman’s rho correlations between ItFNAT scores and rsEEG coherence across three networks (rows) and three frequency bands (columns). Each circular layout depicts electrode positions following the standard 10–20 montage, with lines indicating coherence correlations between electrode pairs. Line thickness reflects the magnitude of the correlation, and line color corresponds to its direction and strength, as shown by the accompanying color scale (blue = negative correlations; red = positive correlations). None of the displayed associations remained significant after FDR correction; the figure illustrates the examined network configurations

Due to significant deviations from normality in several coherence values and ItFNAT scores, three separate sets of nine Spearman’s rho correlations were computed to examine the relationships between ItFNAT scores and network-specific rsEEG coherence. None of the memory–coherence correlations remained significant after Benjamini–Hochberg FDR correction applied separately within each frequency-band family. The smallest FDR-adjusted p values were 0.066 for three low-gamma associations (Table 4; Fig. 4).

**Table 4:** Spearman’s correlations between ItFNAT performance and network-specific resting-state EEG coherence across theta, low-gamma, and high-gamma bands

		Theta	Frontal-parietal	Central-parietal	Low-gamma	Frontal-parietal	Temporal-parietal	High-gamma	Frontal-parietal	Temporal-parietal
		Frontal-midline			Frontal-temporal			Frontal-temporal		
Immediate recall score	Spearman’s ρ	− 0.089	− 0.114	0.021	− 0.235	− 0.233	− 0.349	− 0.149	− 0.091	− 0.217
	P-value	0.627	0.534	0.910	0.196	0.200	0.051	0.417	0.619	0.233
	FDR	0.806	0.801	0.910	0.225	0.225	0.110	0.536	0.696	0.441
	Fisher’s z	− 0.089	− 0.115	0.021	− 0.239	− 0.237	− 0.364	− 0.150	− 0.092	− 0.221
	CI Low	− 0.421	− 0.464	− 0.336	− 0.531	− 0.556	− 0.616	− 0.469	− 0.464	− 0.511
	CI High	0.288	0.256	0.393	0.135	0.170	− 0.019	0.228	0.277	0.098
Delayed free recall score	Spearman’s ρ	− 0.249	− 0.358	− 0.154	− 0.307	− 0.415	− 0.403	− 0.329	− 0.175	− 0.343
	P-value	0.169	0.044	0.399	0.087	0.018	0.022	0.066	0.339	0.055
	FDR	0.761	0.396	0.801	0.131	0.066	0.066	0.297	0.509	0.297
	Fisher’s z	− 0.254	− 0.375	− 0.155	− 0.318	− 0.442	− 0.428	− 0.342	− 0.176	− 0.357
	CI low	− 0.583	− 0.630	− 0.506	− 0.594	− 0.683	− 0.688	− 0.615	− 0.632	− 0.629
	CI high	0.128	0.015	0.271	0.051	− 0.064	− 0.072	0.034	0.245	− 0.016
Delayed total recall score	Spearman’s ρ	− 0.123	− 0.161	− 0.041	− 0.063	− 0.415	− 0.335	− 0.052	− 0.288	− 0.211
	P-value	0.504	0.379	0.823	0.730	0.018	0.061	0.777	0.110	0.245
	FDR	0.801	0.801	0.910	0.730	0.066	0.110	0.777	0.330	0.441
	Fisher’s z	− 0.123	− 0.162	− 0.041	− 0.063	− 0.442	− 0.349	− 0.052	− 0.296	− 0.215
	CI low	− 0.464	− 0.486	− 0.417	− 0.399	− 0.712	− 0.656	− 0.401	− 0.625	− 0.523
	CI high	0.244	0.194	0.331	0.299	− 0.041	0.041	0.301	0.094	0.126

CI Low and CI High represent 95% bootstrap confidence intervals for Spearman’s ρ based on 1000 resamples

Spearman’s ρ, unadjusted and FDR-adjusted p values, Fisher’s z, and bootstrapped 95% confidence intervals (CI) are reported for each association

## Discussion

This study aimed primarily to explore whether resting-state oscillatory dynamics, particularly in the theta (θ) and gamma (γ) bands, were associated with individual differences in face-name associative memory performance as measured by the ItFNAT. This extends our previous work on the link between rsEEG and memory performances (Manippa et al. 2025b) by focusing on associative memory and its electrophysiological correlates. Although significant associations were observed for both low-γ (DTR) and high-γ (IR and DTR) models before correction for multiple comparisons, only the regression model predicting DTR from resting-state high-γ activity remained statistically significant after FDR correction, showing positive associations with frontal and temporal h-γ power and a negative association with posterior h-γ power.

Understanding γ activity requires considering a phenomenon that is not uniform, but that must be examined in terms of its sub-bands, its location, and its network organization. To further explore the hypothesis, γ has been split into two sub-bands, l-γ (30–49 Hz) and h-γ (51–100 Hz). Although significant associations emerged for both γ sub-bands before multiple-comparisons correction, only the high-γ model predicting DTR performance remained significant after FDR correction, highlighting a more prominent role of this frequency range. DTR scores exhibited a similar pattern, with higher frontal and temporal h-γ power and lower posterior h-γ power associated with better performance. These results, confirmed by extensive bootstrapping, accounted for a substantial portion of variance (Adj. R^2^ ~ 38%). This specific role of γ has been well-documented in the literature, highlighting its function in long-term episodic memory (Griffiths and Jensen 2023). High-gamma activity in the medial temporal cortex is associated with the coordination of neural activity within memory-related networks, contributing to both encoding and storage processes (Nyhus and Curran 2010; Kucewicz et al. 2017). Similarly, more sustained spontaneous prefrontal γ activity may reflect executive functions that support memory retrieval. Studies on cognitive control indicate that the frontal lobe plays a role in organizing and retrieving information, particularly in tasks that require significant cognitive effort (Cho et al. 2006; Roux and Uhlhaas 2014). Other studies have shown that both θ and γ power increase in the frontal and temporal areas during memory tasks, indicating greater involvement of attentional and information maintenance mechanisms; this increase may reflect the engagement of the neurophysiological mechanism of attention or WM, suggesting that frontal γ activity reflects the allocation of cognitive resources necessary for binding and associative operations (Ahn et al. 2022; Manippa et al. 2025b).

Posterior high-gamma power showed an inverse association with delayed associative memory performance. Although task-based studies have linked posterior gamma activity to visual and perceptual processing (Puszta et al. 2018), these findings cannot be directly transferred to an eyes-closed resting-state condition, in which no visual task or external sensory interference was present. Therefore, the present negative association should not be interpreted as evidence of sensory-interference suppression. One possible explanation is that posterior high-gamma power reflects individual differences in baseline cortical excitability or excitatory–inhibitory balance, as spontaneous gamma activity has been associated with variations in cortical excitation and inhibition (Grent-’t-Jong et al. 2018a). However, the present study did not directly measure excitatory–inhibitory balance, and the functional meaning of the negative posterior association remains uncertain.

The coherence analyses yielded negative trend-wise associations between delayed-recall performance and resting-state connectivity. Specifically, lower frontal-parietal low-gamma coherence was associated with higher DFR and DTR scores, while lower temporal-parietal low-gamma coherence was associated with higher DFR scores. Although none of these associations survived FDR correction, their negative association with ItFNAT scores differed from task-based studies that generally report increased gamma synchronization during associative learning, short-term memory processing, encoding, and retrieval (Miltner et al. 1999; Babiloni et al. 2004, 2006). The present results should therefore not be interpreted as confirming the task-based gamma-coherence literature. One possible interpretation is that resting-state and task-related coherence may reflect different functional states, with lower baseline coupling potentially permitting greater subsequent network reconfiguration when cognitive demands arise. However, the present study did not measure task-related changes in connectivity or network reconfiguration and therefore cannot directly test this mechanism. These negative resting-state coherence associations should be considered exploratory findings requiring independent replication.

## Future directions, limitations, and conclusions

This study investigated the association between rsEEG θ and γ dynamics and associative memory performance. The regional pattern of γ-power associations broadly resembles that observed in Manippa et al. (2025b), while extending those findings to DTR in a face-name associative memory task and to a broader EEG analytical framework incorporating functional connectivity analyses. In contrast, the exploratory coherence analyses yielded negative associations between delayed-recall performance and frontal-parietal and temporal-parietal connectivity that did not survive FDR correction.

Although no a priori power analysis was conducted, the post-hoc analysis suggests sensitivity only to very large effects, whereas small-to-moderate associations may have remained undetected, affecting the interpretation of the nonsignificant models. Larger independent samples, including more heterogeneous age and clinical groups, are required to estimate smaller effects and assess the generalizability of the present findings.

Finally, the observational and correlational design precludes causal inferences. Although resting-state EEG was recorded before the memory task, temporal precedence alone is insufficient to establish causality, as unmeasured factors may have influenced both resting-state neural activity and memory performance. Accordingly, all reported associations, including those identified in the network-coherence analyses, should be interpreted as statistical relationships requiring replication in larger independent samples. Moreover, because the network-coherence analyses were exploratory and no directional hypotheses were prespecified, the interpretation that lower resting-state coherence may reflect greater network flexibility or facilitate subsequent task-related reconfiguration remains speculative, particularly since task-related connectivity was not measured.

In conclusion, this study offers a two-dimensional examination of rsEEG spectral power density and connectivity in relation to associative memory performance. The results highlight the association between spontaneous fast oscillations and associative memory processes, adding to the growing interest in non-invasive multimodal approaches describing the brain dynamics underlying healthy and pathological aging (Rossini et al. 2020; Cesnaite et al. 2023; Scianatico et al. 2024), providing a rationale for further investigation of stimulation protocols in both healthy and clinical populations (Manippa et al. 2022, 2024; Koch et al. 2024). These findings should be considered specific to healthy young adults and require replication in larger independent samples. Future studies should determine whether the observed associations generalize across age groups and to populations with cognitive impairment before their potential clinical or neuromodulatory relevance can be established.

## Data Availability

The data supporting the results of this study are not publicly available due to privacy and participant protection considerations. However, can be requested from the corresponding author (Giorgia Francesca Scaramuzzi, g.scaramuzzi6@phd.uniba.it) and access will be granted upon approval, in accordance with applicable ethical and legal standards.
